# Targeted Molecular Iron Oxide Contrast Agents for Imaging Atherosclerotic Plaque

**DOI:** 10.7150/ntno.44712

**Published:** 2020-05-30

**Authors:** Rhiannon J. Evans, Begoña Lavin, Alkystis Phinikaridou, Kok Yean Chooi, Zahra Mohri, Eunice Wong, Joseph J. Boyle, Rob Krams, René Botnar, Nicholas J. Long

**Affiliations:** 1 Department of Chemistry, MSRH Building, Imperial College London, White City Campus, 80 Wood Lane, White City, London, W12 0BZ, UK.; 2 School of Biomedical Engineering and Imaging Science, St. Thomas's Hospital, King's College London, London, SE1 7EH, UK.; 3 Department of Bioengineering, Imperial College London, South Kensington, London, SW7 2AZ, UK.; 4 National Heart and Lung Institute, ICTEM Building, Imperial College London, Hammersmith Campus, Du Cane Rd, London, W12 0NN, UK.

**Keywords:** MRI contrast agents, vulnerable plaque, atherosclerosis, superparamagnetic iron oxide nanoparticles

## Abstract

**Overview:** Cardiovascular disease remains a leading cause of death worldwide, with vulnerable plaque rupture the underlying cause of many heart attacks and strokes. Much research is focused on identifying an imaging biomarker to differentiate stable and vulnerable plaque. Magnetic Resonance Imaging (MRI) is a non-ionising and non-invasive imaging modality with excellent soft tissue contrast. However, MRI has relatively low sensitivity (micromolar) for contrast agent detection compared to nuclear imaging techniques. There is also an increasing emphasis on developing MRI probes that are not based on gadolinium chelates because of increasing concerns over associated systemic toxicity and deposits^1^. To address the sensitivity and safety concerns of gadolinium this project focused on the development of a high relaxivity probe based on superparamagnetic iron oxide nanoparticles for the imaging of atherosclerotic plaque with MRI. With development, this may facilitate differentiating stable and vulnerable plaque *in vivo.*

**Aim:** To develop a range of MRI contrast agents based on superparamagnetic iron oxide nanoparticles (SPIONs), and test them in a murine model of advanced atherosclerosis.

**Methods:** Nanoparticles of four core sizes were synthesised by thermal decomposition and coated with poly(maleicanhydride-alt-1-octadecene) (PMAO), poly(ethyleneimine) (PEI) or alendronate, then characterised for core size, hydrodynamic size, surface potential and relaxivity. On the basis of these results, one candidate was selected for further studies. *In vivo* studies using 10 nm PMAO-coated SPIONs were performed in *ApoE*^-/-^ mice fed a western diet and instrumented with a perivascular cuff on the left carotid artery. Control *ApoE*^-/-^ mice were fed a normal chow diet and were not instrumented. Mice were scanned on a 3T MR scanner (Philips Achieva) with the novel SPION contrast agent, and an elastin-targeted gadolinium agent that was shown previously to enable visualisation of plaque burden. Histological analysis was undertaken to confirm imaging findings through staining for macrophages, CX3CL1, elastin, tropoelastin, and iron.

**Results:** The lead SPION agent consisted of a 10 nm iron oxide core with poly(maleicanhydride-alt-1-octadecene), (-36.21 mV, r^2^ 18.806 mmol^-1^/s^-1^). The irregular faceting of the iron oxide core resulted in high relaxivity and the PMAO provided a foundation for further functionalisation on surface -COOH groups. The properties of the contrast agent, including the negative surface charge and hydrodynamic size, were designed to maximise circulation time and evade rapid clearance through the renal system or phagocytosis.* In vitro* testing showed that the SPION agent was non-toxic.

*In vivo* results show that the novel contrast agent accumulates in similar vascular regions to a gadolinium-based contrast agent (Gd-ESMA) targeted to elastin, which accumulates in plaque. There was a significant difference in SPION signal between the instrumented and the contralateral non-instrumented vessels in diseased mice (p = 0.0411, student's t-test), and between the instrumented diseased vessel and control vessels (p = 0.0043, 0.0022, student's t-test). There was no significant difference between the uptake of either contrast agent between stable and vulnerable plaques (p = 0.3225, student's t-test). Histological verification was used to identify plaques, and Berlin Blue staining confirmed the presence of nanoparticle deposits within vulnerable plaques and co-localisation with macrophages.

**Conclusion:** This work presents a new MRI contrast agent for atherosclerosis which uses an under-explored surface ligand, demonstrating promising properties for *in vivo* behaviour, is still in circulation 24 hours post-injection with limited liver uptake, and shows good accumulation in a murine plaque model.

## Introduction

Cardiovascular disease (CVD), including myocardial infarction and stroke, remains a leading cause of death and long-term disability worldwide 2, costing the UK in excess of €26 billion per year to treat 3. The high mortality is mainly caused by coronary atherosclerosis and the high morbidity by carotid atherosclerosis and associated stroke. Atherosclerosis is a chronic, inflammatory, lipid-driven disease of the artery walls. Atherosclerotic plaques are classified in a variety of ways and here we adopt the simple difference between vulnerable and stable plaques, which is based on their propensity to rupture with ensuing thrombosis that might occlude the lumen.

A major challenge for medical imaging is the development of a contrast agent which can differentiate between vulnerable and stable plaques, to identify rupture-prone lesions and perhaps to enable treatment to trigger plaque stabilisation or regression and prevent subsequent cardiovascular events. This is particularly important given the asymptomatic nature of vulnerable plaque, and the low survival rates for out-of-hospital cardiac arrests (as measured by patients subsequently discharged alive from hospital) remaining below 10% 3.

The current gold-standard for human atherosclerosis imaging is Optical Coherence Tomography (OCT) which can differentiate between plaque phenotypes, but which is an invasive technique. Computer tomography has also shown great promise in vulnerable plaque detection but exposes patients to inonizing radiation and thus is not ideal for repeat exams 4.

Magnetic Resonance Imaging (MRI) is a non-invasive imaging technique with excellent resolution which does not use ionising radiation to generate excellent soft tissue contrast. To further enhance contrast between normal and diseased tissues, or to obtain molecular and cellular information, MR contrast agents can be used. By far the majority of MRI contrast agents are based on gadolinium-chelates which generate positive T_1_ contrast through paramagnetism. However, increasingly, alternatives to gadolinium are being sought due to toxicity concerns particularly in patients with kidney disease, and recent studies which have found gadolinium deposits in patients' brains 1,5,6.

Commercial, off-the-shelf, non-targeted SPIONs have been previously used to image atherosclerotic plaques in patients 7,8. Whilst these studies indicate that SPIONs successfully image atherosclerotic plaques, the exact correlation with plaque phenotype is less clear.

The aim of this study was to develop a range of MRI contrast agents based on superparamagnetic iron oxide nanoparticles (SPIONs) with different surface functionalisation, and test them in a murine model of advanced atherosclerosis to determine whether a long-circulating, non-toxic SPION contrast agent might offer an alternative to invasive intravascular OCT or ultrasound, and to more traditional gadolinium-containing MRI contrast agents for the detection of vulnerable plaque.

## Results and Discussion

### Contrast agent development

SPIONs were synthesised using the thermal decomposition method first reported by Sun et. al. 9. Four different size cores (6, 8, 10, 12 nm) were grown to examine the relationship between core size and relaxivity. The hydrophobic particles were phase transferred using three different ligand systems, poly (maleic anhydride-alt-1-octadecene) (PMAO) 10, alendronate 11, and poly(ethyleneimine) (PEI). The surface ligands all offered different characteristics, comparing encapsulation (PMAO), and ligand exchange (alendronate and PEI), different surface groups (-COOH and -NH_2_), as well as newer (PMAO) and more established ligands (PEI).

The nanoparticles were characterised in terms of core size, hydrodynamic size, surface charge, and relaxivity. Hydrodynamic size and surface charge are both important considerations in predicting and governing *in vivo* behaviour. Long circulation times allow the contrast agent to accumulate in plaque through a combination of phagocytosis by plaque macrophages, and the enhanced permeability and retention (EPR) effect arising from endothelial dysfunction. This would also provide a platform for potential targeting of the probe to vulnerable plaque-specific proteins such as CX3CL1 12-16, VCAM-1 17-19, VEGF 20, or αvβ3 integrin 21 through antibodies, which require long-circulation to be effective targeting moieties. In addition to an imaging platform, targeting the probe to chemokines such as CX3CL1, CCR2 or CCL5 would have potential therapeutic benefits. These chemokines are all associated with vulnerable plaque, and blocking their expression has been shown to lead to plaque stabilisation and regression 15,22. Developing a long-circulating probe targeted to one or multiple of these proteins would aid in the detection of vulnerable plaque, as well as treating it *in situ* and improving patient outcomes.

**Figure 1:**
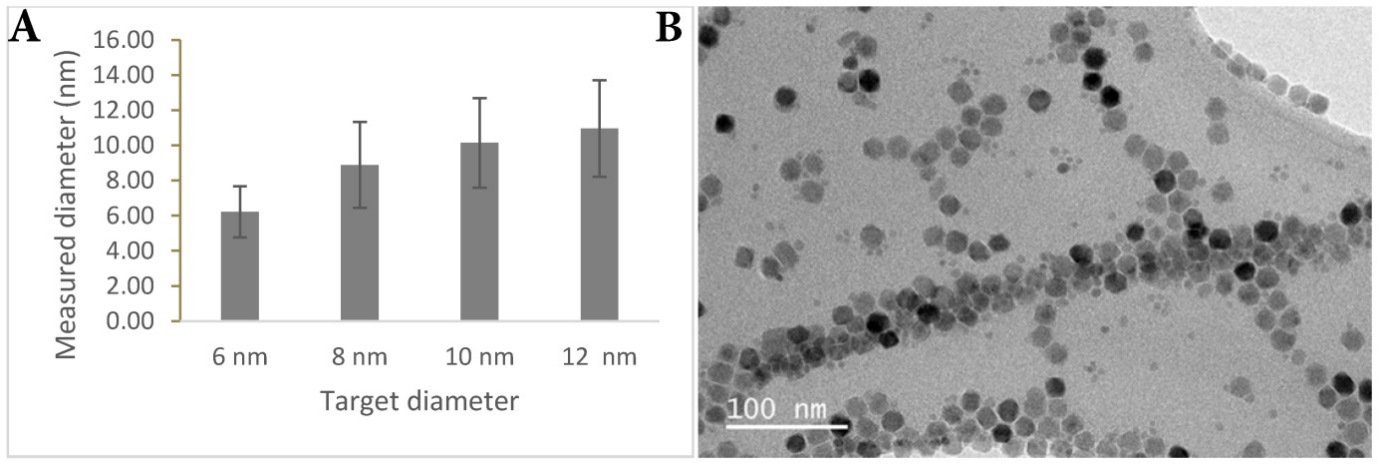
** TEM characterisation. (A)** Graph showing measured nanoparticle core size versus projected nanoparticle core size. **(B)** TEM image showing irregular faceting of nanoparticle cores.

In order to ensure the long circulation of the probe, the clearance route was of primary consideration. There is a size window between 6-200 nm for avoiding renal clearance (<6 nm) 23 and clearance immediately through the reticuloendothelial system (>200 nm). Core size was measured by transmission electron microscopy (TEM) (**Figure 1**) and hydrodynamic size through dynamic light scattering (DLS) (**Table 1**). Surface charge particularly affects interaction with the immune system, where neutral agents are longer-circulating, positively-charged agents clear faster due to higher intracellular uptake resulting from the electrostatic attraction to the cell membrane, and opsonisation by proteins in the blood stream accelerating phagocytosis 24-26. Negatively-charged agents are not as long-circulating as neutral agents but are better than positively-charged agents for antibody targeting.

DLS and visual inspection both showed the formation of large aggregates with both amine-based surface ligands, and PEI in particular, was very challenging to exchange onto the nanoparticles despite testing multiple conditions. PMAO showed much narrower size dispersions and improved stability, in water and phosphate buffered saline in accordance with literature findings 27. The carboxylic acid groups on the surface of the PMAO-coated particles resulted in a negative surface charge, and that in combination with the narrower size dispersion, lack of aggregation and long-term stability made it the most suitable choice for this contrast agent. PMAO has also not seen application *in vivo* yet, and the probe is therefore novel in surface functionalisation, and looks to be a promising platform for many applications. Nanoparticle contrast agents are easily tuned to many different targets and applications, and -COOH groups are easily functionalised with targeting moieties for molecular imaging, dyes or fluorophores for optical imaging and potentially histology, chelators for radionuclides or gadolinium, meaning that this probe has potential across a wide spectrum of applications and modalities.

The relaxivity measurements were the decisive factor in selecting the lead-candidate for antibody-coupling, with the 10 nm nanoparticle cores showing the highest r_2_ (18.806 mmol^-1^s^-1^). An anti-CX3CL1 antibody was coupled to the surface of the probe through carbodiimide coupling to test the feasibility of molecular targeting, and tests indicated that the antibody was successfully coupled to the nanoparticle surface, and that it retained binding ability after coupling.

### *In vitro* evaluation

**Figure 2:**
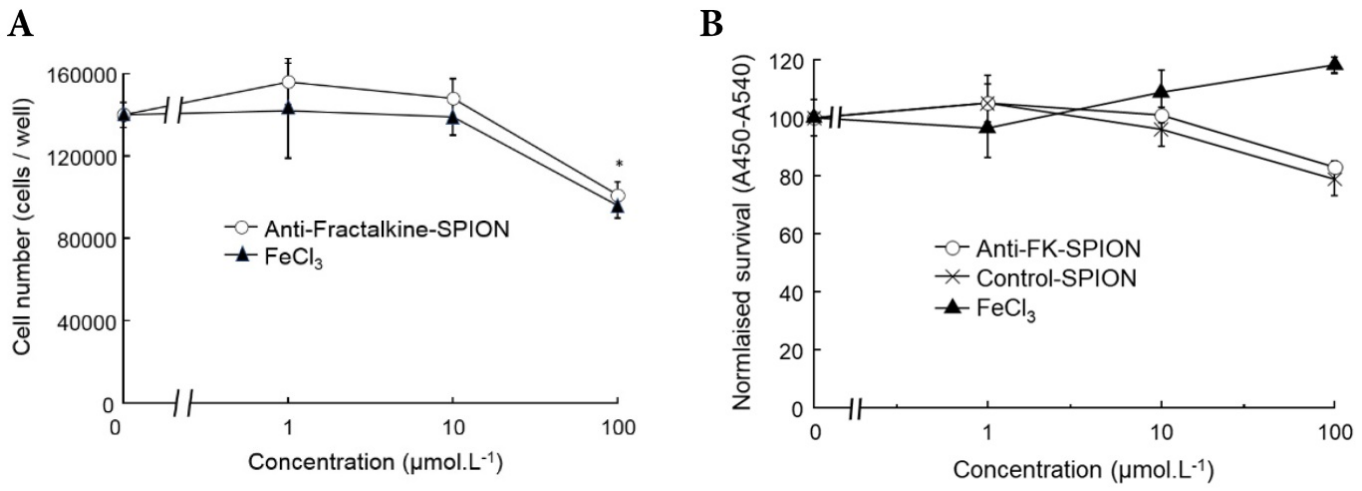
** Cell survival assays with SPIONS.** Graphs demonstrating measured cell survival of RAW 264.7 murine macrophages at 24 hours incubation with varying concentrations of iron in the form of anti-Fractalkine SPIONs or FeCl_3_. **(A)** Cell counts using a hemacytometer at 24 hours showing an equivalent and slight loss of cells at 24 hours at extreme concentrations (100 µM) of SPIONs and FeCl_3_. **(B)** Colorimetric cell survival assay based on formazan (XTT) reduction, also carried out at 24 hours and also showing an equivalent slight loss of cells at 24 hours at extreme concentrations (100 µM) of SPIONs, whether targeted or untargeted (no antibody). FeCl_3_ increases apparent cell number, although that is likely to represent an artefact e.g. mitochondrial dysfunction. Data were also obtained at 2 hours, when there was very little effect (not shown).

A cell viability assay using RAW 264.7 murine macrophages was undertaken to confirm the contrast agent was non-toxic. Cells were incubated with varying concentrations of iron in excess of what might be encountered *in vivo*, and the SPIONs were compared to FeCl_3_. Cell viability was assessed using a standard tetrazolium/formazan cell viability dye [2,3-bis-(2-methoxy, 4-nitro, 5-(sulfophenyl)-2H-tetrazolium-5-carboxanilide) (XTT)] measured with absorbance spectroscopy, by minor modification of previous methods 28-32. Higher measured cell survival was observed for those cells treated with SPIONs as opposed to FeCl_3_ at all concentrations, as can be observed in Figure 2. For each concentration, cell survival was higher for SPIONs than the corresponding amount of FeCl_3,_ further evidence that the SPIONs were at least as well tolerated as unencapsulated Fe. For reference, Fe is measurable in peripheral serum at a peak concentration of approximately 10 mM after a conventional dietary iron supplement 33 (**Figure 2**).

**Figure 3:**
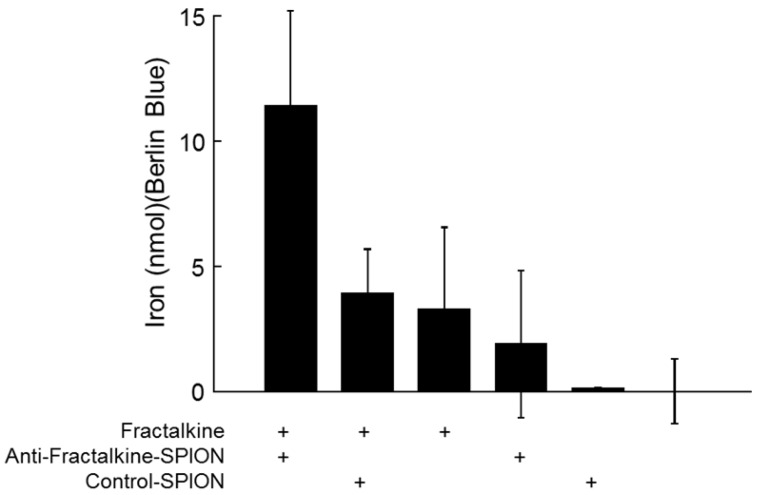
** Specific binding of anti-factalkine SPIONs to immobilised fractalkine shown by Berlin-Blue based iron measurement.** Graph showing bound as detected by Berlin Blue and UV-Vis. spectroscopy in wells coated with CX3CL1 and incubated with targeted and non-targeted SPIONs. *p<0.05, ANOVA.

In order to confirm that the anti-fractalkine-SPION conjugate specifically targeted fractalkine, a binding assay was designed. Both targeted and non-targeted SPIONs were incubated against murine CX3CL1 or control-coatings on 96-well plates (see Methods). To obtain an unambiguous demonstration that iron-containing particles were captured by plate-adsorbed fractalkine, unbound particles were removed through a gentle wash and plate-bound iron detected a reaction product with Berlin Blue (acidified potassium ferricyanide, a classical reagent for detecting immobilized iron) added. After 10-20 mins incubation, Berlin Blue/iron complex was measured by UV-Vis. spectroscopy. The results (**Figure 3**) demonstrated higher levels of bound iron detected in the wells with targeted SPIONs as opposed to control SPIONs.

### *In vivo* evaluation

The contrast agent was tested in a murine model of advanced (vulnerable) plaque: 6 *ApoE^-/-^* C57/Bl6 female mice fed a high fat diet with a cuff placed on the left carotid artery for 9 weeks to cause a stenosis and changes in wall shear stress patterns. The model was developed to cause a vulnerable plaque upstream of the cuff and a stable plaque downstream of the cuff, which made it particularly suitable for testing this contrast agent by enabling the direct comparison of uptake in known vulnerable and stable plaque sites 34.

**Figure 4:**
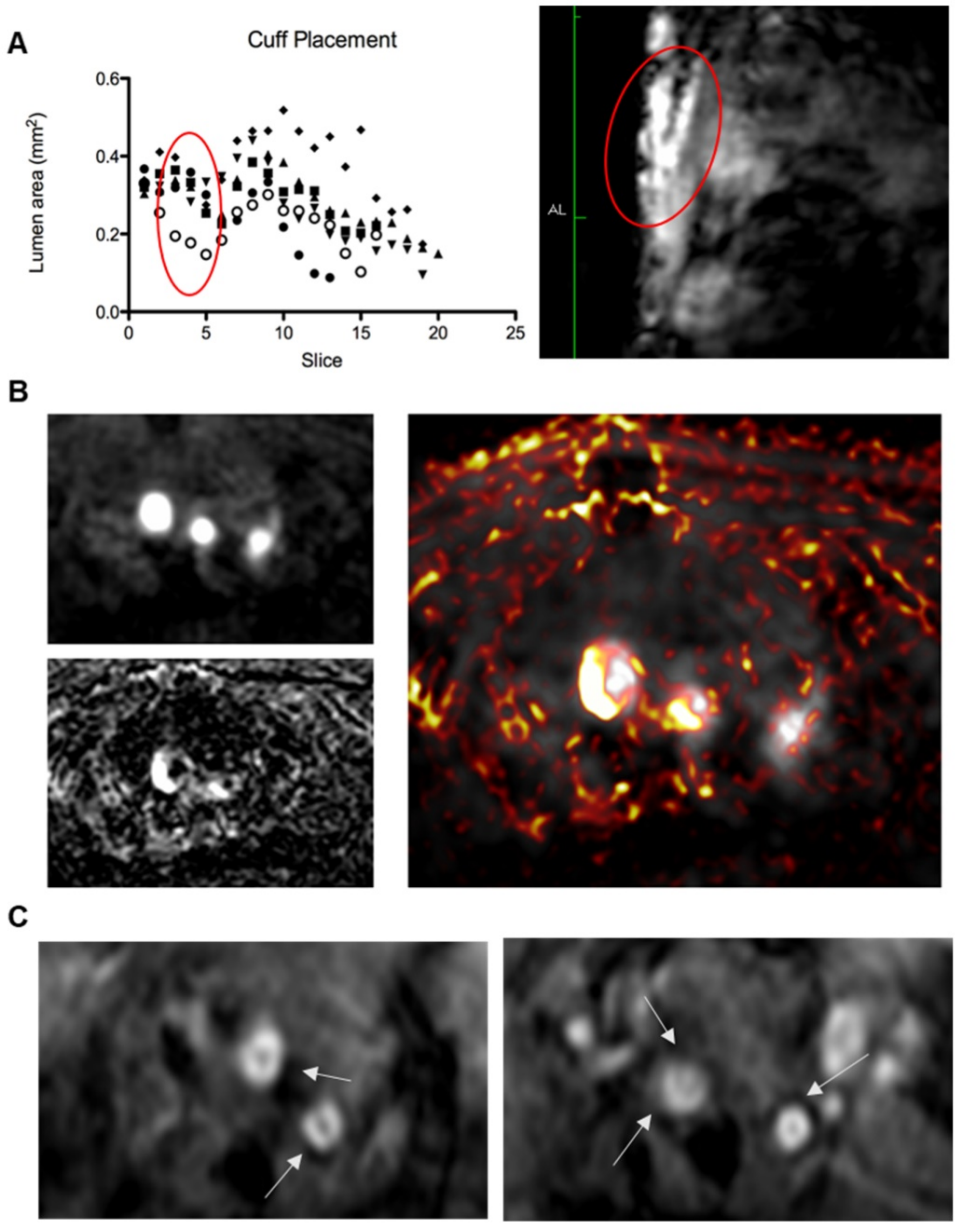
Top to bottom, **(A)** Left: graph showing lumen area versus slice number, demonstrating a consistent decrease in lumen area from slice 3-5 (standardised relative to aortic arch) in all 6 animals (represented by different legends) corresponding to cuff location; right: anatomical scan showing the location of the cuff and resulting arterial stenosis; **(B)** Left: Magnetic Resonance Angiography scan (Top) showing the brachiocephalic artery, left carotid artery, and left subclavian artery (L-R), Bottom: Inversion recovery image showing plaque areas with Late Gadolinium enhancement from Gd-ESMA in the vessel walls. Right: Image overlay showing the areas of enhancement in relation to the vessel lumen. **(C)** Two examples of T_2_^*^ echo scans based on the protocol from Makowski et. al. 35 showing negative contrast areas in the vessel walls.

**Figure 5:**
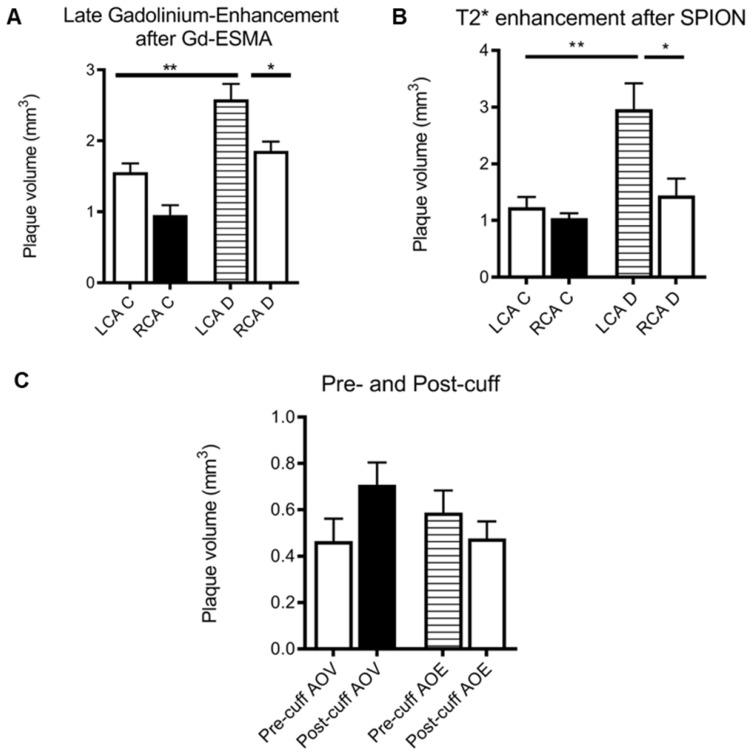
** (A)** Graph showing plaque volume as measured by area of late-gadolinium enhancement in both the left (LCA) and right (RCA) carotid arteries of control (left) and diseased (right) animals, showing that cuff placement and hyperlipidemia increase plaque size in the instrumented diseased left carotid artery (*p<0.05, student's t-test); **(B)** graph showing plaque volume as measured by T2* enhancement in the left (LCA) and right (RCA) carotid arteries of control (left) and diseased (right) animals, showing that cuff placement hyperlipidemia increase plaque size in the instrumented diseased left carotid artery (*p<0.05, student's t-test); **(C)** graph showing area of void (AOV) arising from SPIONs and area of late-gadolinium enhancement (AOE) arising from Gd-ESMA in control regions pre- (upstream) and post-cuff (downstream), although differences are present, no level of significance was reached.

The mice were first injected with a gadolinium-containing elastin-specific MR contrast agent (Gd-ESMA) which has been characterised in atherosclerosis models, allowed to visualise the location and extend of plaque and provided a benchmark for the novel contrast agent. The mice were then injected with the SPIONs and imaged 24 hours post-injection. The results are illustrated in **Figures 4 and 5**.

The Gd-ESMA scans showed a significant difference between uptake in the cuffed vessel compared with the contralateral control vessel (p = 0.018), demonstrating that the cuff placement and hyperlipidemia led to the formation of plaque more rapidly than hyperlipidemia alone through alterations of wall shear stress. There was also a significant difference between the Gd-ESMA signal in the cuffed disease vessel compared to both the left and right carotid arteries in the control animals ((p = 0.002, 0.000095), supporting the plaque formation as a result of hyperlipidemia and placement of the cuff with resultant stenosis (**Figure 5**). The data was analysed using multiple non-parametric t-tests corrected for multiple comparisons by the Holm-Sidak method, and was found to be statistically significant. There was no notable difference in Gd-ESMA signal between the stable and vulnerable plaque regions measured in three slices above and below the stenosis of the cuff, but it was not expected that elastin content would differ significantly, particularly given that Gd-ESMA binds to both the cross-linked mature form of elastin, and its precursor tropoelastin. Interestingly, the brachiocephalic artery was observed to be enlarged related to other murine atherosclerosis models, and this is likely to be a result of compensation due to the restriction the cuff places on the left carotid.

The SPION behavior mirrored Gd-ESMA signal, validating the novel contrast agent against an established agent to measure plaque burden. Again, there was a significant difference between the modified vessel and the internal control vessel (p = 0.018), and between the modified, diseased vessel and both vessels in the control animalsp = 0.005, 0.002) (**Figure 5**). The data was analysed using multiple non-parametric t-tests corrected for multiple comparisons by the Holm-Sidak method, and was found to be statistically significant.

There was no significant difference between the uptake of either contrast agent between the vulnerable plaque upstream of the cuff, and the stable plaque downstream of the cuff (p = 0.32, student's t-test) (**Figure 5**).

### Histological verification

**Figure 6:**
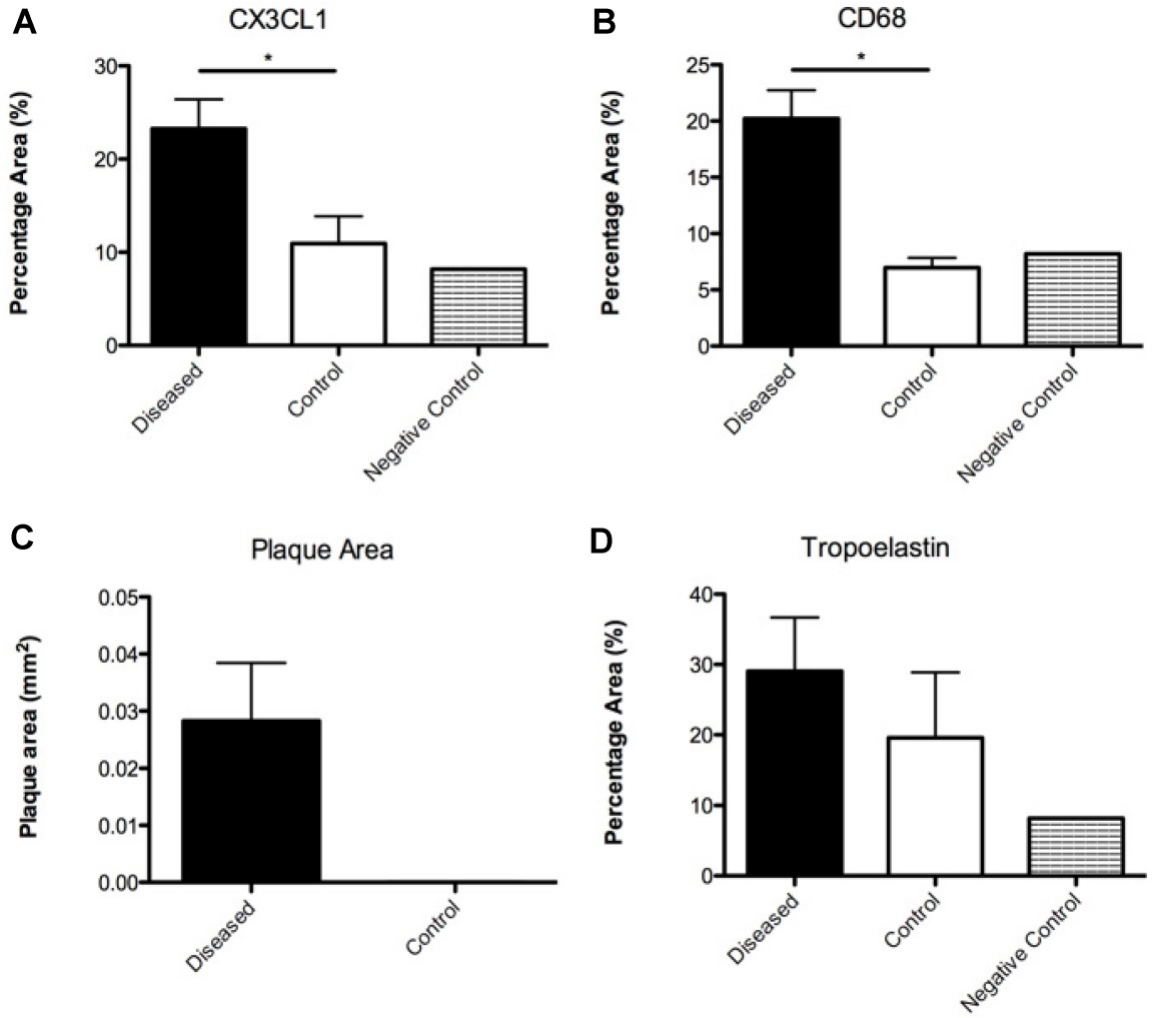
From top to bottom and left to right: **(A)** Graph illustrating the percentage vessel wall area of CX3CL1 measured in diseased and control sections; **(B)** Graph illustrating the percentage vessel wall area of CD68 measured in diseased and control sections; **(C)** Graph illustrating the plaque area measured in diseased and control sections; **(D)** Graph illustrating the percentage vessel wall area of tropoelastin measured in diseased and control sections.

**Figure 7:**
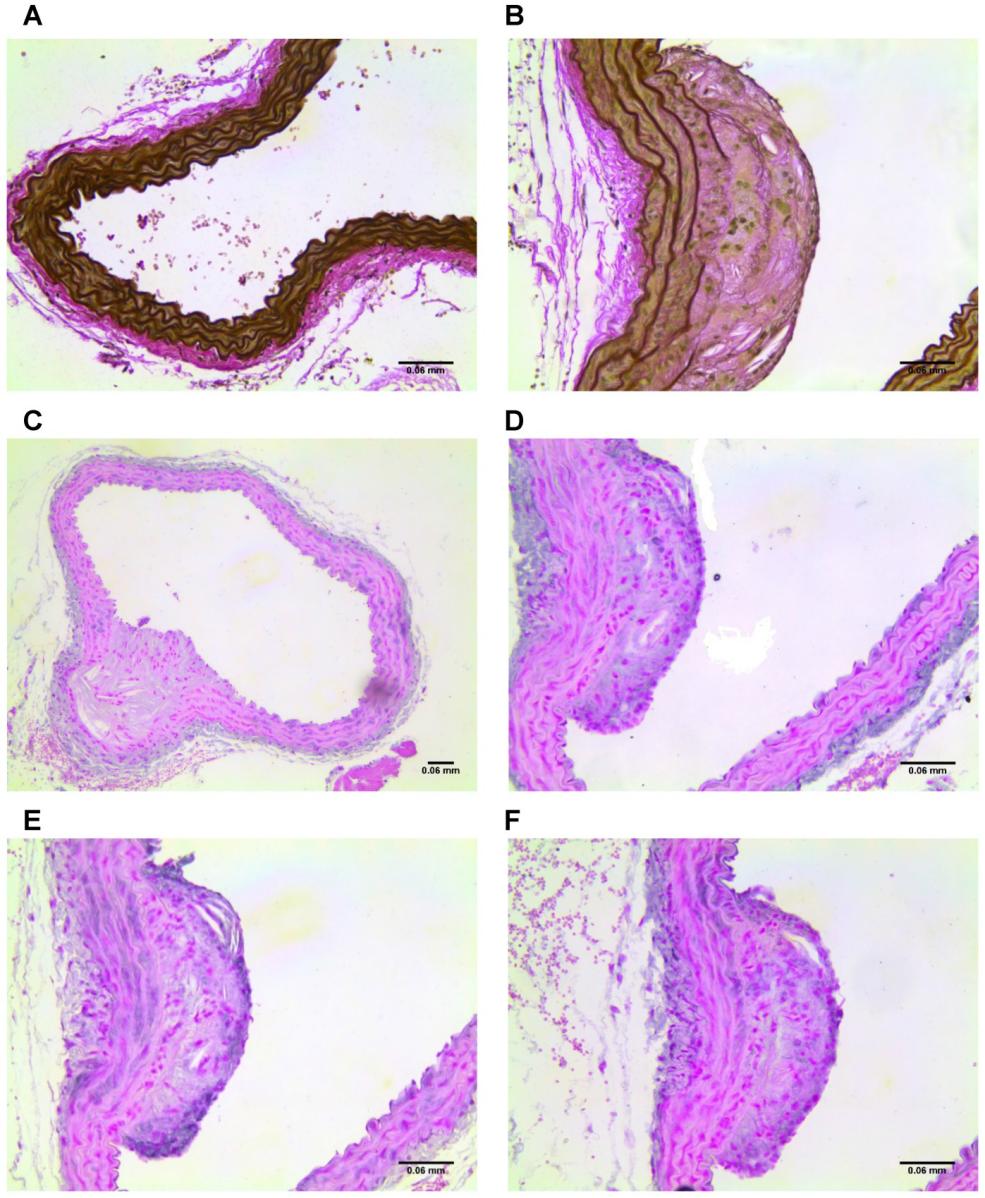
** (A)** Section of control vessel stained for elastin and, **(B)** section of diseased vessel stained for elastin showing a plaque. **(C-D)** Sections of diseased vessel stained for tropoelastin, showing a plaque. **(E)** Section of diseased vessel showing a plaque stained for CD68, and **(F)** section of diseased vessel showing a plaque stained for CX3CL1.

**Figure 8:**
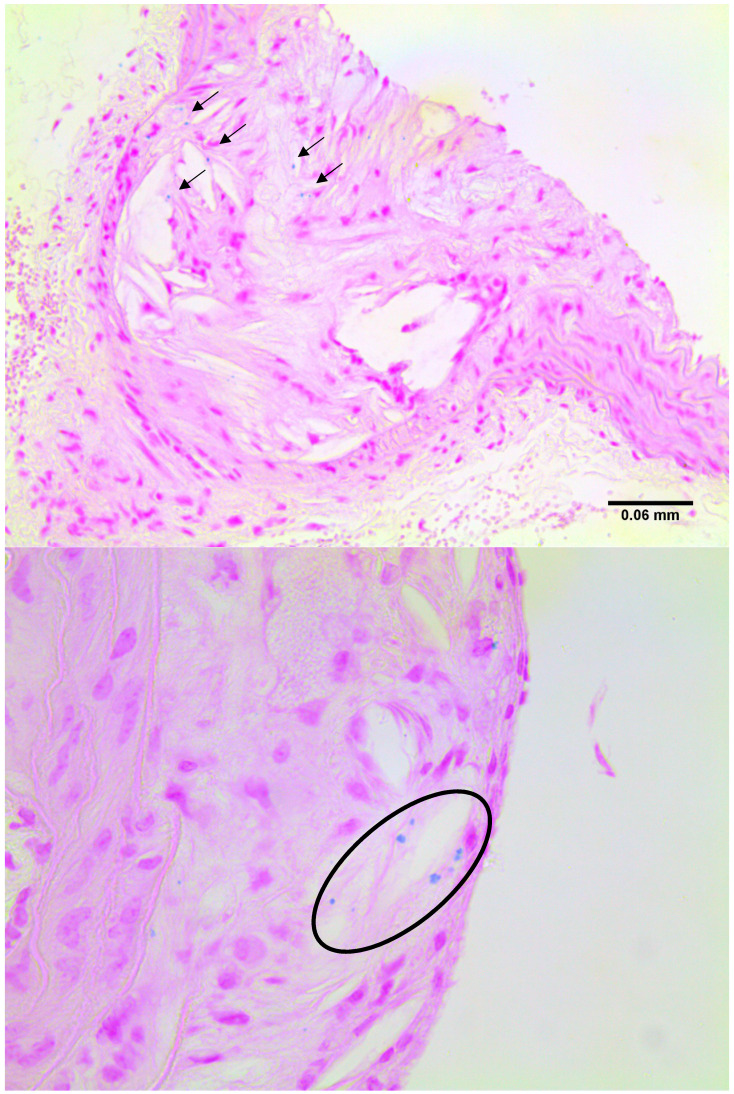
Examples of sections stained for iron with Berlin Blue, highlighting the blue deposits in plaque regions.

Imaged plaques and control sections of artery were then analysed through histology and immunohistochemistry, staining sections for elastin, tropoelastin, CD68, CX3CL1 (a marker for vulnerable plaque), and iron. Berlin blue staining for iron confirmed the presence of SPION-derived iron in the plaques of diseased vessels (Figure 8), but there was no Berlin blue staining in the control vessels. There was a significant difference in expression of both CD68 which is a macrophage marker, and Fractalkine between sections of tissue from diseased and healthy control animals. Levels of tropoelastin were also elevated in plaque regions. These findings are illustrated in **Figures 6 and 7**.

## Conclusions

In conclusion, this paper illustrates the development of a novel superparamagnetic iron oxide nanoparticle contrast agent which successfully allowed imaging of atherosclerotic plaques with MRI. Although the probe is not specific for vulnerable plaque, it is non-toxic and accumulates in similar regions to an elastin-targeted agent for visualizing plaque burden. It also provides a good platform for further refinement towards a vulnerable plaque-specific contrast agent in the future. The novel probe has successfully been labelled with an antibody, and the conjugated antibody has been demonstrated to retain binding specificity for the target. There is a limitation in that the nanoparticles were labelled with an anti-CX3CL1 antibody, however this labelling was at a low proportion of the injected dose and so the probe has not been treated as a targeted agent. Because of this, no conclusions can be drawn regarding potential levels of CX3CL1 expression in the plaques, although previous work by Cheng et. al. suggests that CX3CL1 is elevated in vulnerable plaques 11.

## Methods

### Chemistry

#### Synthesis of 6 nm hydrophobic SPIONs 36

Fe(acac)_3_ (0.71 g, 2 mmol) and 1,2-hexadecanediol (2.58 g, 10 mmol) were dissolved in benzyl ether (20 ml). Oleic acid (2.11 ml, 6 mmol) and oleylamine (2.80 ml, 6 mmol) were added, and the mixture was stirred for 2 hours under N_2_ at 200 °C. The mixture was then heated to reflux (300 °C) for 1 hour and cooled by the removal of the heat source.

Nanoparticles were precipitated with EtOH (40 ml) and centrifuged for 30 minutes at 4000 rpm. Pellet was re-dispersed in hexane (20 ml), centrifuged for 10 minutes at 4000 rpm and then precipitated again with EtOH (20 ml). The solution was centrifuged for 30 minutes at 4000 rpm, supernatant removed and pellet dried under vacuum. SPIONs were stored dry.

#### Seed-mediated growth of SPIONs 36

Fe(acac)_3_ (0.18 g, 0.5 mmol) and 1,2-hexadecanediol (0.65 g, 2.5 mmol) were dissolved in benzyl ether (15 ml). Oleic acid (0.70 ml, 0.5 mmol), oleylamine (0.93 ml, 0.5 mmol) and SPIONs (5-10 mg) were added and the mixture was stirred for 1 hour at 200 °C. The mixture was then heated to reflux (300 °C) for half an hour and cooled by the removal of the heat source.

The workup was performed as with 6 nm hydrophobic SPIONs.

#### Coating with poly(maleic anhydride-alt-1-octadecene) 10

Poly(maleic anhydride-alt-1-octadecene) (PMAO) (45 mg) was dissolved in CHCl_3_ (20 ml). The SPIONs (2 mg) were added and mixture was stirred for 30 minutes. Chloroform was removed, and residue re-dissolved in a small amount of CHCl_3_. NaOH (aq) (0.05 M) was added and mixture was heated at 60 °C until SPIONs transferred to the aqueous phase.

The solution was filtered using on a 0.45 µm filter, and then concentrated by centrifugation with a filter of 30k Mw. Particles were re-suspended in water and stored.

#### Coating with alendronate 11

Sodium alendronate trihydrate (50 mg) was dissolved in water (5 ml) adjusted to pH 9 with 1 M KOH. The SPIONs (6 mg) were suspended in THF. The solutions were combined and stirred at room temperature for 2 days. The solution was magnetically decanted and washed with acetone (3 × 10 ml). The particles were re-suspended in water and stored.

### Biology

#### *In vitro* cell toxicity assay

##### Preparation of XTT working reagent

5 ml of PBS (Phosphate Buffered Saline) and 50 µl of XTT (25 mg/ml) and 0.5 µl PMS (Phenazine methosulfate, 50 mg/ml) was added into a clean 15 ml conical centrifuge tube.

In a 96 well plate each well was seeded with 200 µl of 10k RAW 264.7 cells in IMDM with 10% FBS. The plate was incubated overnight, and then treated with SPIONs at different concentrations: 0 µM, 1 µM, 10 µM and 100 µM. The plate was incubated overnight, and 20 µl of XTT reagent as prepared above was added to the wells and incubated. The absorbance was measured at 2 and 24 hours post-addition of XTT at 450 and 540 nm.

#### Animal Model

All animal experiments complied with the Animals (Scientific Procedures) Act 1986 and were carried out under PPL 70/8482 at King's College London.

Homozygous female ApoE-/- mice (C57BL/6J background) were acquired from Charles Rivers Laboratories (Edinburgh, UK) and bred within the Behavioural Sciences Unit of the Rayne Institute. The housing and care of the animals, and all procedures in this study were performed in accordance with the guidelines and regulations of the UK home office.

At 11 weeks of age the mice were placed on high fat diet (HFD) (21% fat, 0.25% cholesterol wt/wt from LBS-Biotech) for two weeks prior to surgery. Two weeks after commencement of HFD the mice underwent surgery for the placement of a rigid polyether ketone perivascular cuff (Promolding, The Netherlands) around the left carotid. The cuff was supplied in two pieces and when assembled the lumen narrowed from 500 µm to 250 µm over 1.5 mm. The animals were kept under anaesthesia at 1.5-2.5% isoflurane and 2% O_2_, with anaesthetic depth being assessed from respiratory rate, hind limb muscle tone, and pedal withdrawal reflex as previously described 34.

The animals then remained on HFD for a further 9 weeks before scanning and sacrifice. Animals were euthanised under Schedule 1 with an overdose of anaesthetic followed by exsanguination and flushing of the vasculature with saline.

Healthy ApoE-/- mice with no cuff fed a normal chow diet were used as controls.

#### Imaging Studies: Gd-ESMA

Based on previous studies carried out by Makowski et. al. 37 animals were injected with 0.2 mmol/kg Gd-ESMA in 150 µl sterile saline through the tail vein. The animals were then scanned 1 hour post-injection.

*In vivo* vessel wall imaging was performed using a Philips Achieva MR scanner (Philips Healthcare, Best, The Netherlands) equipped with a clinical gradient system (30 mT m^-1^, 200 mT m^-1^ ms^-1^).

The brachiocephalic artery (BCA) and left and right carotids were imaged using a single-loop surface coil (diameter = 23 mm). Mice were placed in prone position after intravenous administration of 0.2 mmol/kg Gd-ESMA (Lantheus Medical Imaging, North Billerica, MA). Anesthesia was induced with 5% and maintained with 1% to 2% isoflurane during the MRI experiments.

After a 3-dimensional gradient recalled echo scout scan, contrast-enhanced angiography images were acquired with a field of view (FOV) = 30× 30× 8 mm, matrix = 200× 200, in-plane resolution = 0.15× 0.15× 0.5 mm (reconstructed = 0.10× 0.10 mm), repetition time/echo time = 15/6.1 ms, and flip angle = 40°. The maximum intensity projection images were used to plan the subsequent delayed-enhancement (DE) and T_1_ mapping scans. A 2-dimensional Look-Locker sequence was acquired using the following parameters: FOV = 30× 30 mm, matrix = 80× 80, in-plane resolution = 0.38× 0.38× 2 mm, repetition time/echo time = 19/8.6 ms, repetition time between subsequent inversion recovery pulses = 1000 ms, and flip angle = 10°. An inversion recovery 3-dimensional fast gradient echo sequence was acquired with FOV = 30× 30× 8 mm, matrix = 304× 304, in-plane resolution = 0.1× 0.1× 0.5 mm, repetition time/echo time = 28/8 ms, repetition time between subsequent inversion recovery pulses = 1000 ms, and flip angle = 30°. T_1_ mapping was performed with the same sequence employed for the aorta. The acquisition parameters were as follows: FOV = 36× 22× 8 mm, matrix = 192× 102, in-plane resolution = 0.18× 0.22× 0.5 mm, slices = 16, repetition time/echo time = 9.6/4.9 ms, flip angle = 10°.

#### Imaging Studies: SPIONs

Animals were injected with 150 µl 6 mM [Fe] SPIONs in sterile saline through the tail vein. The animals were then scanned 24 hours post-injection.

The imaging protocols were based on previous experiments by Makowski et. al. 35 and were carried out on a Phillips 3T Achieva scanner (Philips Healthcare, Best, The Netherlands) equipped with a clinical gradient system (30 mT m^-1^, 200 mT m^-1^ ms^-1^) as before.

The brachiocephalic artery (BCA) and left and right carotids were imaged using a single-loop surface coil (diameter=23 mm). Mice were placed in prone position. Anesthesia was induced with 5% and maintained with 1% to 2% isoflurane during the MRI experiments.

After a 3-dimensional gradient recalled echo scout scan, contrast-enhanced angiography images were acquired with a field of view (FOV) = 30× 30× 8 mm, matrix = 200× 200, in-plane resolution = 0.15× 0.15× 0.5 mm (reconstructed = 0.10× 0.10 mm), repetition time/echo time = 15/6.1 ms, and flip angle = 40°. The maximum intensity projection images were used to plan the subsequent T_2*_-weighted 3D gradient echo scan. ECG-triggering was not used, and a T_2*_-weighted 3D gradient echo data set was acquired with FOV = 16× 16× 8; matrix = 176× 176; in-plane spatial resolution = 0.12× 0.12 mm; slice thickness = 0.5 mm; TR/TE = 37.88/7.32 ms and flip angle = 25°.

### Histology

#### Elastin Staining

Deparaffinised slides were incubated in working elastic stain solution for 10 minutes and rinsed in deionized water. Slides were then placed in ferric chloride solution for 22 seconds, rinsed under running water and checked under the microscope. Slides were rinsed in 96% ethanol to remove excess iodine, then rinsed in deionized water. Slides were incubated in van Gieson solution for 1-3 minutes. Finally, slides were dehydrated through a gradient of ethanol, cleared in xylene, and mounted.

#### Prussian Blue Staining

20% aqueous solution of hydrochloric acid (20 ml conc. HCl, 80 ml water) and 10% potassium ferrocyanide trihydrate (10 g, 100 ml water) were prepared and mixed fresh before staining. Slides were immersed in the solution for 20 minutes then washed in deionized water three times before counterstaining with nuclear fast red for 5 minutes. Finally, slides were dehydrated through a gradient of ethanol, cleared in xylene, and mounted.

#### Immunohistochemistry

##### Deparaffinised slides were stained for tropoelastin, CD68 or CX3CL1 using the following protocol

Endogenous peroxidase was inhibited by incubation of the slides in 3% oxygenated water and methanol for 10 minutes. Slides were then washed in deionized water for 10 minutes. Antigen retrieval was performed by boiling the samples for 2 minutes at 110 °C in citrate buffer in a pressurized antigen retrieval unit and then allowed to cool. Samples were washed with PBS for 3 minutes, and blocking was performed for 1 hour at room temperature with 10% donkey serum in PBS. Incubation with the primary antibody was carried out overnight at 4 ˚C diluted 1:100 in PBS 2% normal donkey serum. Samples were allowed to temper at room temperature for 1 hour, then slides were washed twice in PBS for 20 minutes. Slides were incubated with anti-rabbit HRP polymer for 45 minutes at room temperature then washed 3 times in PBS for 5 minutes.

Slides were revealed with Vector SG Peroxidase substrate for 10 minutes at room temperaturePBS (5 ml), Chromogen (75 μl), Hydrogen peroxide (120 μl).

Samples were washed twice in PBS for 5 minutes, before counterstaining with nuclear fast red for 5 minutes. Slides were washed with deionized water then finally dehydrated through a gradient of ethanol, cleared in xylene, and mounted.

## Supplementary Material

Supplementary figure S1.

## Figures and Tables

**Table 1:** Hydrodynamic size and surface potential measurements for all synthesised nanoparticles

Sample	Diameter in nm	Potential in mV
6 nm iron oxide with PEI	148.5	3.30
8 nm iron oxide with PEI	206.5	1.60
10 nm iron oxide with PEI	---	10.91
12 nm iron oxide with PEI	371.5	5.73
6 nm iron oxide with PMAO	113.2	-42.11
8 nm iron oxide with PMAO	104.7	-36.66
10 nm iron oxide with PMAO	194.6	-36.21
12 nm iron oxide with PMAO	139.6	-22.84
6 nm iron oxide with alendronate	199.5	26.86
8 nm iron oxide with alendronate	292.7	12.12
10 nm iron oxide with alendronate	194.0	26.60
12 nm iron oxide with alendronate	266.4	28.44
