# Supplementary material for: Targeted Molecular Iron Oxide Contrast Agents for Imaging Atherosclerotic Plaque
Source: Nanotheranostics. 2020 May 30;4(4):184–94. doi: 10.7150/ntno.44712 (PMC7332796; doi:10.7150/ntno.44712)
Supplement: Supplementary file 1 — Supplementary figure S1. [file ntnov04p0184s1.pdf]

Electronic Supporting Information

Targeted Molecular Iron Oxide Contrast Agents for Imaging Atherosclerotic Plaque

Rhiannon J. Evans, Begoña Lavin, Alkystis Phinikaridou, Kok Yean Chooi, Zahra Mohri, Eunice Wong, Joseph J. Boyle, Rob Krams, René Botnar, Nicholas J. Long

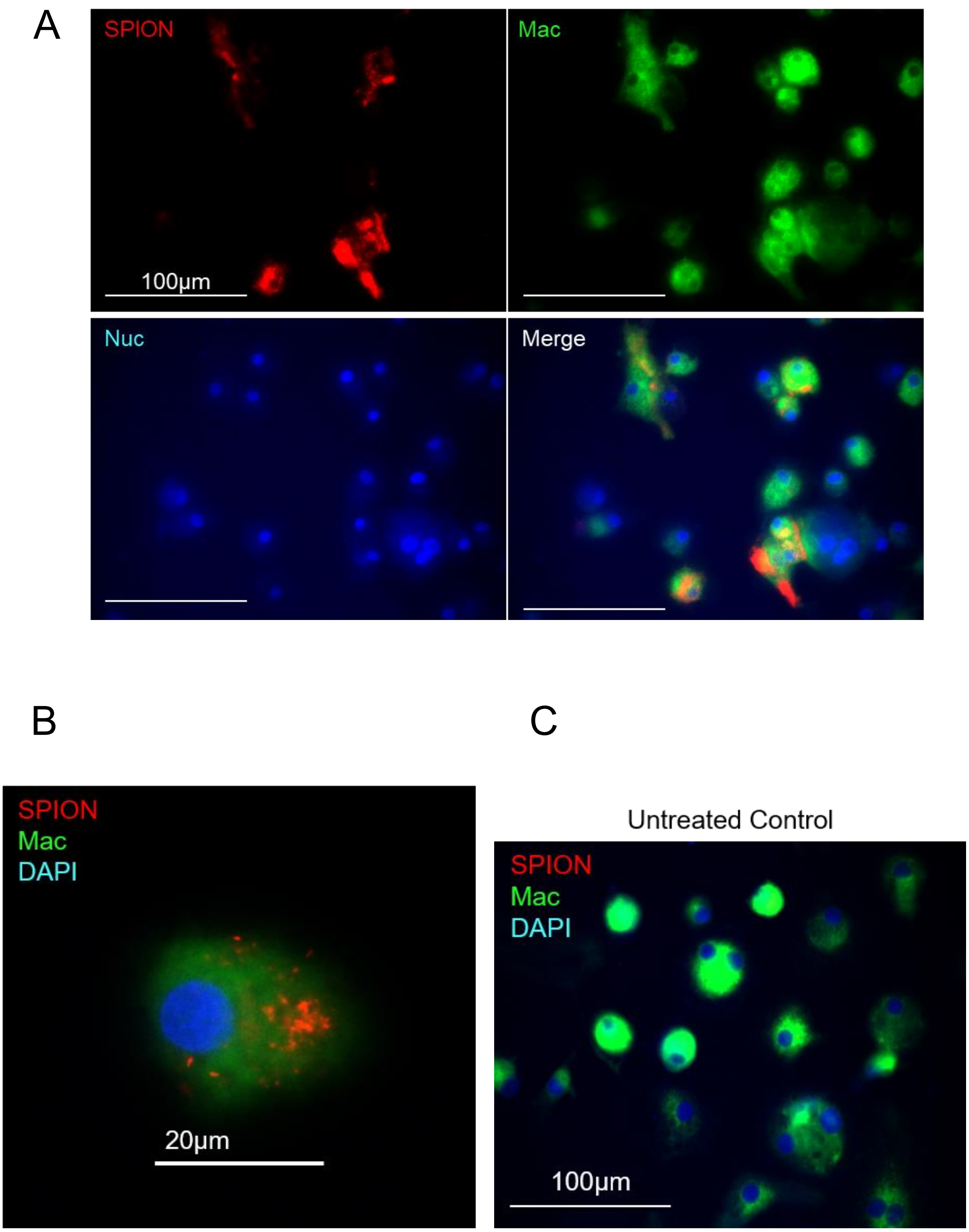

Supporting Figure 1:

Fluorescence microscopy identifies SPION within macrophages. Anti-FK-SPION were incubated with anti-rabbit secondary Alexa-568 at 1:1 molar concentration. RAW macrophages were dyes with the stable lipophilic cell tracer Di-O. The labelled SPIONs were then incubated in slide chambers at 37 ^{o}C for 30 min. Hoechst, a cell-permeant nuclear DNA-binding dye was added for 5 mins, the cells were fixed in 1% formaldehyde 5 mins at 4 ^{o}C and then imaged by 4-channel fluorescence microscope (Leica DM2500).

A. typical image at x40. The overlay is shown. Colours are true colour (RGB camera). Scalebar – indicated distance. Rd and yellow within the merge image – SPION within macrophages.
B. High magnification clearly showing SPION within macrophages.
C. Specificity, lack of SPION-associated fluorescence in macrophages incubated without SPION.
